# ABG Assistant—Towards an Understanding of Complex Acid-Base Disorders

**DOI:** 10.3390/jcm10071516

**Published:** 2021-04-05

**Authors:** Łukasz Gutowski, Kaja Gutowska, Alicja Brożek, Marcin Nowicki, Dorota Formanowicz

**Affiliations:** 1 Department of Medical Chemistry and Laboratory Medicine, Poznan University of Medical Sciences, Rokietnicka 8, 60-806 Poznan, Poland; lgutowski@ump.edu.pl (Ł.G.); abrozek@ump.edu.pl (A.B.); patogen@gmail.com (M.N.); 2 Institute of Computing Science, Poznan University of Technology, Piotrowo 2, 60-965 Poznan, Poland; Kaja.Gutowska@cs.put.poznan.pl

**Keywords:** arterial blood gas, acid-base, mixed disorders, ABG algorithm, corrected AG, evaluation of acid-base disorders, acidosis, alkalosis, prediction, diagnostics, personalized patient profiling, ABG application

## Abstract

The ability to diagnose acid-base imbalances correctly is essential for physicians and other healthcare workers. Despite its importance, it is often considered too complex and confusing. Although most people dealing with arterial blood gases (ABGs) do not usually have problems with acid-base disorder assessment, such an analysis is also carried out by other healthcare workers for whom this can be a challenging task. Many aspects may be problematic, partly due to multiple data analysis methods and no definitive statement on which one is better. According to our survey, the correctness of arterial blood gas analysis is unsatisfactory, especially in mixed disorders, which do not always manifest an obvious set of symptoms. Therefore, ABG parameters can be used as an established biomarker panel, which is considered to be a powerful tool for personalized medicine. Moreover, using different approaches to analyze acid-base disorders can lead to varying diagnoses in some cases. Because of these problems, we developed a mobile application that can spot diagnostic differences by taking into account physiological and chemical approaches, including their variants, with a corrected anion gap. The proposed application is characterized by a high percentage of correct analyses and can be an essential aid for diagnosing acid-base disturbances.

## 1. Introduction

Blood gas analysis is an essential diagnostic tool in emergency departments and for clinical management under many conditions. Most often arterial blood is used for such analysis. However, venous blood gas (VBG) is sometimes analyzed because of infection at the insertion site or inadequate collateral circulation of extremities, In some specific clinical conditions, VBG can even be interpreted as interchangeable with arterial blood gas (ABG) [1]. Arterial blood gas (ABG) analysis gives essential information about the patient’s oxygenation status and Acid-Base balance. Further analysis requires much more data: electrolytes, lactate, glucose, osmolality, creatinine, and urine. However, even limiting the analyses only to the data obtained from ABGs causes difficulties for medical staff.

The problem of incorrect diagnosis of Acid-Base disorders starts at the universities. Different approaches and equations for secondary responses are taught at various universities because there is no agreement on which ABG analysis approach gives the best results [2,3]. Additionally, even within particular approaches, the terminology is not standardized. Despite the growing need for standardization and the establishment of appropriate standards [4] there is no consensus on ABG analysis or the teaching of it, which leads to difficulties in further learning. A student who has been taught a specific ABG analysis approach, while trying to expand his or her knowledge, collides with different terminology and approaches to the topic and has to spend much more time comprehending the differences and similarities among the other methods. If a physician starts working in a place where ABG is often analyzed, he or she adapts to the particular approach and terminology. With extended practice, such a person usually uses all possible analysis methods interchangeably and does not have any problems with terminology, such as when exchanging information about the case with other hospital staff. However, in practice, blood gases are rarely analyzed, so these skills are not intensively developed. For these healthcare workers, whether they be physicians, medical laboratory scientists, or paramedics, programs that help analyze patient results can be useful.

As mentioned above, complications, like multiple secondary-response equations, can lead to different diagnoses. Moreover, the analysis of mixed disorders, accompanied by an increasing number of coexisting disturbances, carries with it a higher risk of error. Because the mentioned equations are not accurate, they raise many concerns [5]. Similar reservations apply to the ratio between the serum anion gap (AG) change and the change in the bicarbonate (HCO_3_^−^) concentration (ΔAG/ΔHCO_3_^−^ ratio) predictability, which depends on the factor causing the disorder and can be a false positive or negative [4,6,7,8,9]. 

The use of the AG enables a more precise analysis of the metabolic acidosis causes. Because AG precision is limited, Figge et al. [9] proposed adding the influence of hypo- or hyperalbuminemia into the AG calculations. However, the analyzers do not calculate the corrected anion gap (AGc), so healthcare workers have to do this by themselves. Thus, when analyzing ABG, it is necessary to perform many calculations, often in a short time. It can be a cumbersome task and the cause of many mistakes.

Given the issues considered, we decided to develop a mobile application that not only analyzes data and checks its correctness but also compares the compatibility of diagnoses in four ways: (1) using the physiological (Boston) approach, (2) the physiological approach with the corrected anion gap, (3) the chemical (Copenhagen) approach, and (4) the chemical approach with the AGc [10,11,12,13]. The summary of these analyses is intended to show whether the diagnosis, previously made by the medics, is confirmed or if the patient’s results are in a so-called grey area, which means that he can be diagnosed differently depending on the concept used. In this case, a signal was given that special attention should be paid while analyzing such a patient.

The application can be downloaded free of charge from the Google Play Store website [14], and its source code is available at GitHub [15]. Although programs supporting the analysis of Acid-Base disorders are not a new idea [16,17], in recent years we have witnessed the development of ABG analysis approaches [10,11,12,18,19,20] and technological progress; hence, we propose our ABG Assistant. Our application uses newer compensation formulas, anion gap ranges adapted to modern analyzers, and appropriate AG correction depending on the albumin concentration change. The developed program is based on existing ABG analysis algorithms; however, it has been improved by sets of parameters causing ambiguous diagnoses. Additionally, a function analyzing mixed disorders with less noticeable metabolic acidosis and unified division of disorders due to the value of the ΔAG/ΔHCO_3_^−^ ratio was introduced.

Since most people use smartphones daily, our program has been written in the Java programming language as an application for the Android system.

## 2. Materials and Methods 

### 2.1. Description of the Application

The activities package contains six activities corresponding to the adequate application windows. The activity responsible for data entry also includes the code’s main part—analyzing the entered parameters. This activity is described in more detail in Section 2.2, “The Algorithm”. After performing the analyses using particular approaches, the results were transferred to the activity responsible for displaying the results. There, the compliance of the diagnosis was calculated. If the application finds that any of the approaches were not performed, it informs the user why it happened, which data was not entered.

**Figure 1:**
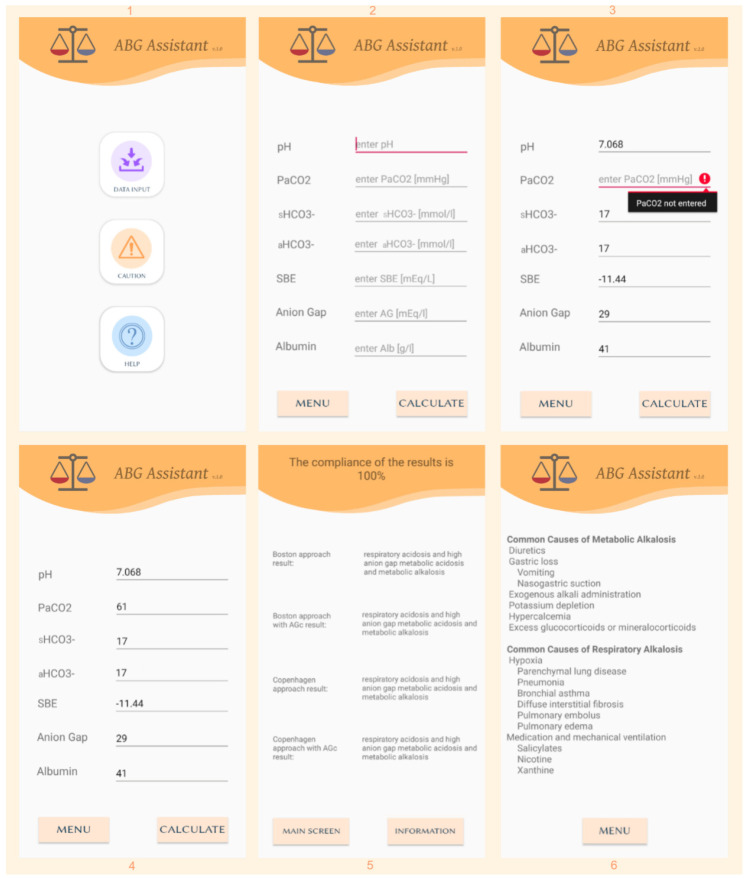
The individual screens of the ABG Assistant showing: (1) menu, (2) data input screen, (3) example warning if required data is not entered, (4) input of sample data, (5) results for previously entered data, (6) fragment of “information” screen.

For the application’s proper functioning, a mobile device with a minimum screen resolution of 720 × 1280 px, equipped with Android version 5.0 or later, is required. The program consists of six windows. The first main window has three buttons: “data input”, “caution”, and “help” (Figure 1). The “caution” button leads to a window where the user is warned about the restrictions on using the application. First of all, the diagnosis should be based on comparing the patient’s clinical condition and history with laboratory results—one cannot rely solely on ABG Assistant analysis. Another button—“help” leads to a window where the application’s basic operating principles are described. The “data input” button leads to the central part of the program. After clicking on it, a list of parameters that can be entered, along with the appropriate units, is displayed. The program has been designed to analyze results based on four possible variants simultaneously; however, some parameters do not have to be entered; without them, the program will analyze data using fewer concepts. The program was intended to work with standardized parameters, like standard HCO_3_^−^ (sHCO_3_^−^) and Standard Base Excess (SBE). However, it is not possible to obtain standardized parameters for every blood gas analyzer. Even though the use of actual HCO_3_^−^ (aHCO_3_^−^) might be misleading in respiratory disorders, in many cases it is still used regularly, so actual HCO_3_^−^ may be used for sHCO_3_^−^ in the main part of the application. The program always needs pH, sHCO_3_^−^, partial pressure of carbon dioxide (PaCO_2_), and an AG to run. Without albumin (Alb), the program will not correct the anion gap, providing results for 2 out of 4 available methods. If the user does not enter SBE, the program will calculate it based on pH and aHCO_3_^−^ (if provided). However, due to the multitude of formulas for SBE [13,21,22], the data may not be identical to those the ABG analyzer would give. After clicking the “calculate” button, the program will open the window with analyses that could be carried out using the entered data. Information describing how many approaches have been used is displayed at the top of the screen, indicating the results’ conformity. Below are the results of the particular methods. If a user does not enter some of the parameters, the corresponding information is displayed. The application gives a preliminary diagnosis of Acid-Base imbalance by determining if it is acute, chronic, simple, or complex. Users are informed if the entered ABG results are incorrect. The program warns if the difference between the entered and calculated pH is higher than 0.02. It also displays a report stating that the entered data are incorrect if the difference is greater than 0.1. Warnings are also shown for extreme values of entered parameters, which indicates false data. The user can get additional information to help make a diagnosis by clicking on the “information” button. It redirects to a window where the most common causes of Acid-Base disorders are listed.

### 2.2. The Algorithm

The main steps taken by the proposed algorithm are based on the works of Kenrick Berend et al. [11,12,20]; however, several changes have been introduced. Currently, there is no agreement as to what reference ranges should be used [4]. In our article we used the following ranges: pH: 7.34–7.44, PaCO_2_: 36–44 mm Hg, HCO_3_^−^: 22–26 mmol/L, SBE: −2–2 mmol/L, AG: 3–11 mEq/L, Alb 35–50 g/L. In contrast to other applications, our algorithm used a lower AG reference range because higher ones were characteristic for older analyzers. This modest change had a big impact on the calculations’ correctness. According to research, the correct AG reference range should be 3–11 using the market-dominant ion-selective electrode (ISE) [22,23,24]. Certainly, the best solution would be to define a reference range for each patient individually in a healthy state, but this is rarely determined. Hence, the ranges provided by the laboratory are more often used. Some of the applications assessing Acid-Base disorders rely on entering Na and Cl values and calculating the AG instead of directly entering the AG value. However, this unnecessarily forces the user to enter further data as the formula for calculating the anion gap is almost always the same. The AG originates from the equation: Na^+^ K^+^ + unmeasured cations = Cl^−^ + HCO_3_^−^ + unmeasured anions. Therefore, the formula using potassium was previously used: AG = (Na^+^ + K^+^) − (Cl^−^ + HCO_3_^−^). Because the normal potassium reference range is low, the formula has been simplified to AG = Na^+^ − (Cl^−^ + HCO_3_^−^). Most of the studies analyzing the AG refer to this formula. Therefore, for the final analysis there is no difference whether the application allows users to enter the equation’s components or uses an already calculated AG—its value is still the same. 

The next step is calculating the AGc. Correction of the AG is calculated according to the equation: AGc = observed anion gap + 0.25 × (normal albumin concentration − observed albumin concentration) based on publication [10]. Other programs often use data in the form of integers for all values or floating-point numbers only for pH [25,26], thus limiting the accuracy of the analysis. 

Our application was designed to enter the SBE value obtained from the ABG analyzer. However if the SBE is not given, the program estimates it based on aHCO3- and pH using the van Slyke equation: 0.9287 × (HCO_3_^−^ − 24.4 + 14.83 × (pH − 7.4)) [1]. The program checks the entered parameters’ correctness individually and their combined compliance with the Henderson–Hasselbalch equation. The cases we analyzed usually did not exceed the difference between the measured and calculated pH by more than 0.015; however, different ABG analyzers use different measurement methods and formulas for calculating parameters, and this difference may increase, especially in severe clinical cases [27]. Therefore, two restrictions have been introduced: (1) exceeding the difference of 0.02 will trigger a notification that the result may be incorrect, and (2) exceeding the difference by 0.1, will cause the program to display information about incorrect results. The compensation formulas for the physiological method were taken from [19,20] and from [11] for the chemical method. According to the prevailing hypothesis, no disturbance can be fully compensated for; all disorders with correct pH are classified as mixed disorders [12,28]. Instead of the Δ–Δ gap used in the work of Berend et al., the proposed algorithm uses the ΔAG/ΔHCO3- ratio. If metabolic acidosis is detected, the ΔAG/HCO_3_^−^ ratio is used as appropriate for the reference ranges (AG − 7)/(24 − HCO_3_^−^). When the AG is equal to or greater than 25 mEq/L, the program warns the user to consider the presence of additional disorder. When the AG is equal to or greater than 30 mEq/L, there is the certainty of additional high AG metabolic acidosis [29]. In both cases, the ΔAG/ΔHCO_3_^−^ ratio is then checked. There is a lot of controversy about the values at which the ΔAG/ΔHCO_3_^−^ ratio indicates an additional disorder [6,9]. Some authors claimed that the ΔAG/ΔHCO_3_^−^ ratio in high anion gap metabolic acidosis should be exactly equal to 1. Greater than 1 would indicate additional metabolic alkalosis; below 1, an additional, normal anion gap metabolic acidosis. Nevertheless, this approach was poorly compatible with many disorders, especially lactic acidosis [6]. Besides, it should be noted that the ΔAG/ΔHCO_3_^−^ ratio gives many results inconsistent with the actual diseases [9]. Therefore we used the following division for ΔAG/ΔHCO_3_^−^:

- <0.8 indicates high anion gap metabolic acidosis together with possible normal anion gap metabolic acidosis;
- 0.8–1.2 indicates high anion gap metabolic acidosis;
- 1.2–2 indicates high anion gap metabolic acidosis but probably not together with metabolic alkalosis;
- >2 indicates high anion gap metabolic acidosis together with metabolic alkalosis.

Three examples are provided to explain how the algorithm works. The data from Table 1 and an example of correct results were used for the considerations.

**(1)**  **pH, 7.4; PaCO_2_, 40 mm Hg; HCO_3_^−^, 24 mEq/L; SBE, 0; AG, 11 mEq/L (Na, 140 mEq/L; Cl, 105 mEq/L); Alb, 40 g/L.**
  (a) The entered data is analyzed. Based on the AG and the albumin, the AGc is calculated. The amount of data entered permits the use of the four approaches. For such data, all four methods are analyzed in a similar way:
  (b) The algorithm checks the consistency of the entered data. There are no noticeable errors.
  (c) The pH is checked. It is within the reference range.
  (d) PaCO_2_ and HCO_3_^−^ are checked by the methods using the physiological approach, and PaCO_2_ and SBE are examined using the chemical approach. All data are within reference ranges. In this step, rare cases of mixed disturbances and measurement errors are excluded (e.g., HCO_3_^−^ = 40 while the rest of the parameters are within normal limits).
  (e) AG is checked. It is within the reference range. Because all of the values were within the reference ranges, and the algorithm did not find any abnormalities in the entered data, the application will display the result: “correct results” in the fields corresponding to all four approaches and above them: “The compliance of the results is 100%.”
**(2)**  **pH, 7.6; PaCO_2_, 45 mm Hg; HCO_3_^−^, 51 mEq/L; AG, 14 mEq/L.**
  (a) The entered data is analyzed. If the user assumes that the sHCO_3_^−^ and aHCO_3_ values are the same and enters these into the application, the SBE will be calculated. The application will use two approaches—physiological and chemical. Otherwise, the amount of data entered allows using a physiological approach.
  (b) The algorithm checks the consistency of the entered data. There are no noticeable errors.
  (c) The pH is checked. The value exceeds 7.44, which indicates alkalosis.
  (d) PaCO_2_ and HCO_3_^−^ are checked by the method using a physiological approach, and PaCO_2_ and SBE are examined using the chemical approach. PaCO_2_ is above 36 mmHg; hence the program will check to. See if metabolic alkalosis is present. Then the HCO_3_^−^ is checked using a physiological approach, and SBE is checked using a chemical approach. These values are not too low, so there are no errors or mistypes (e.g., a value of HCO_3_^−^ of 10 mmol/L or SBE of −15 would not be not possible given the pH/PaCO_2_ parameters and would indicate erroneous data).
  (e) The algorithm checks the correct range of the secondary response to metabolic alkalosis. In a physiological approach, according to the formula PaCO_2_ = 0.7 × (HCO_3_^−^ − 24) + 40 ± 2 mm Hg. The PaCO_2_ should therefore be in the range of 56.9–60.9 mm Hg. Since the PaCO_2_ value is much lower than expected, there is also respiratory alkalosis. The PaCO_2_ value differs from the calculated one by more than 5%—application displays the message: “metabolic alkalosis and respiratory alkalosis”. However, if the PaCO_2_ value differs from the calculated one by less than 5%, instead of the mentioned response, the application would display the message: “metabolic alkalosis, probably with respiratory acidosis”. In a chemical approach SBE is calculated according to the formula: SBE = 0.9287 × (HCO_3_^−^ − 24.4 + 14.83 × (pH − 7.4)) [20] equals to 27,458.
  (f) According to the formula ΔPaCO_2_ = 0.6 × SBE mm Hg [13]. PaCO_2_ should be equal to 56.475. Since the PaCO_2_ value is much lower than expected, there is also respiratory alkalosis. The PaCO_2_ value differs from the calculated one by more than 5%; therefore, the application displays the message: “metabolic alkalosis and respiratory alkalosis”. However, if the PaCO_2_ value differs from the calculated one by less than 5%, instead of the mentioned response, the application would display the message: “metabolic alkalosis, probably with respiratory acidosis”. Although the AG is elevated, such fluctuations are possible with this type of disorder and are not the basis for suspecting the presence of metabolic acidosis. However, if the AG exceeds 25 mEq/L, the application informs the user that another disorder should be considered. When the AG exceeds 30 mEq/L, the application informs the user about an additional disorder.
  (g) Because the entered values made it possible to analyze the results with the use of 2 approaches, and the algorithm did not find any abnormalities in the data, the application will display the result: “metabolic alkalosis and respiratory alkalosis” in the fields corresponding to both of these approaches, and above them: “Comparing two approaches. The compliance of the results is 100%”.
**(3)**  **pH, 7.06; PaCO_2_, 28 mm Hg; HCO_3_^−^, 8 mEq/L; AG, 10 mEq/L; Alb, 23 g/L.**
  (a) The data is analyzed. AGc is calculated according to equation AGc = observed anion gap + 0.25 x (normal albumin – observed albumin). If the user assumes that the values of sHCO_3_^−^ and aHCO_3_^−^ are the same, and enters these values into the application, the SBE will be calculated. The application will use two approaches—physiological and chemical. Otherwise, the amount of data entered permits the use of a physiological approach.
  (b) The algorithm checks the consistency of the entered data. There are no noticeable errors.
  (c) The pH is checked. The value is lower than 7.36, which indicates acidosis.
  (d) PaCO_2_ and HCO_3_^−^ are checked using a physiological approach, and PaCO_2_ and SBE are examined using a chemical approach. PaCO_2_ is below 44 mm Hg; hence the program will check if metabolic acidosis is present.
  (e) The algorithm checks the correct range of the secondary response for metabolic acidosis in all approaches, analogous to the previous case. Since PaCO_2_ is higher than it should be, additional respiratory acidosis is expected.
  (f) The value of the anion gap is checked:
    - With the use of physiological and chemical approaches without AG correction, the AG is 10 mEq/L, so the algorithm diagnoses normal anion gap metabolic acidosis and respiratory acidosis.
    - With the use of physiological and chemical approaches, the AGc equals 14.25 mEq/L. When using the AGc, the algorithm diagnoses the presence of Hagma, not Nagma, as was suspected using the previous approach. In such an event, the ΔAG/ΔHCO_3_^−^ ratio must also be calculated. Its value is less than 0.8 [7]; hence, apart from the high anion gap metabolic acidosis and respiratory acidosis, normal anion gap metabolic acidosis is also suspected.

As the entered values made it possible to analyze the results using all approaches, the algorithm did not find any abnormalities in the entered data. The application displayed the result: “normal anion gap metabolic acidosis and respiratory acidosis” in the fields corresponding to the approaches without the AGc, and “high anion gap metabolic acidosis and respiratory acidosis, possible additional normal anion gap metabolic acidosis” in the areas corresponding to the approaches using the AGc. Above them, the program displayed, “The compliance of the results is 50%.”

**Figure 2:**
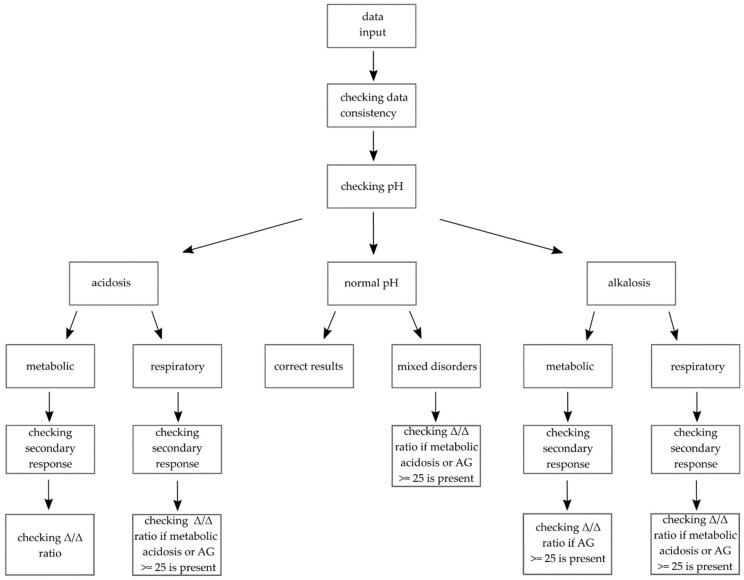
Simplified scheme of the algorithm. When the data is entered, the program analyses its consistency. If there are no obvious errors, the pH is checked. Next, based on the PaCO_2_ and HCO_3_^−^ (or SBE in chemical approach), disorders are categorized as metabolic or respiratory. Then, the secondary response is checked. If there is a primary or additional high anion gap metabolic acidosis present, Δ/Δ ratio is checked to detect possible triple disorders. Δ/Δ ratio is also checked when the AG is equal to or higher than 25 mEq/L.

A simplified scheme of the proposed algorithm is given in Figure 2.

### 2.3. The Survey

To assess the ability to analyze ABGs, we conducted a survey consisting of 10 questions. For each of them, the patient’s results had to be analyzed to assess the Acid-Base disorder. In each question, diagnosing the disorder was limited to a selection from a comprehensive list of possible ones. The data for each case is presented in Table 2, together with the correct analysis. The reference ranges can differ depending on the laboratory or research. Therefore, at the beginning of the questionnaire, the reference ranges that the respondents were obligated to use were presented. These ranges are presented in Section 2.2, “The Algorithm”. The introduction also included all the necessary formulas to estimate the correctness of a secondary response for a given disturbance, the Henderson–Haselbalh equation, a simplified equation for calculating the hydrogen concentration, an ΔAG/ΔHCO_3_^−^ equation, and an equation for the AGc. Thanks to this, we were sure that we only assessed the respondents’ ability to analyze the data and that the reference values and formulas used were identical and did not affect the respondents’ answers. Respondents (healthcare workers: physicians, medical laboratory scientists, and paramedics) could have filled in a paper questionnaire or used the online form.

The questionnaire could also have been completed by students of medical universities, provided that they had already passed ABG analysis training. Before proceeding with the diagnosis of disorders, respondents had to answer questions about their profession or education, professional experience, the approach they use daily, and how often they analyze ABG data. 

## 3. Results

### 3.1. Evaluation

Evaluation of the application was based on a set of ABG tests taken from the medical literature [30] and 10 selected ABG disturbances, which were also used to compare the application and human accuracy of the diagnosis. According to the application’s functionality, the proposed diagnoses of Acid-Base disorders were made using up to four methods: physiological, chemical and variants of these approaches using the AGc. The list of parameters and diagnoses used in the mentioned publication has been compared with the proposed application results and is included in Table 1. The following parameters have been taken into account: pH, partial pressure of carbon dioxide (PaCO_2_), bicarbonate (HCO_3_^−^), standard base excess (SBE), Anion Gap (AG), and albumin (Alb). 

In most cases, the program was consistent with the diagnosis or even more detailed although there were cases in the cited literature [30], where the diagnoses differed slightly. Each of these cases is listed below with an explanation of the difference in the approach to analysis: 

Cases no. 1 and 2—the application gave more detailed results than the literature; it specified the type of metabolic acidosis. 

Case no. 3—in the publication, this case was diagnosed as metabolic acidosis and respiratory acidosis. As mentioned earlier, all diagnoses made solely based on the ABG results can differ depending on the formulas used. Our application also warned about the possibility of the co-occurrence of an additional disorder (“possible additional metabolic alkalosis”) because the data gave results close to the limit value. If the results exceed the calculated compensation range but do not exceed it by 5%, the application says that the values are close to the correct range; however, there is a possibility of the co-existence of an additional disorder.

Partial incompatibility occurred in cases no. 3, 4, and 9. In all these cases, the results were close to the cut-off value, so the program warned about the possibility of additional interference. In case no. 8, a similar situation occurred. Here, the results obtained from the application warned about “the possible additional metabolic alkalosis”, but the cited diagnosis only mentioned the presence of “metabolic alkalosis”.

Case no. 9. Here, the additional value of albumin concentration was given, allowing the use of the application’s full functionality. In this way, the program used two additional methods that calculated the corrected anion gap, thanks to which the results were more precise than when taking into account only one of the methods. As a result, instead of merely obtaining information: “normal anion gap metabolic acidosis and respiratory acidosis,” the application also displayed: “high anion gap metabolic acidosis and respiratory acidosis, possible additional normal anion gap metabolic acidosis.”

### 3.2. Comparison of the Interpretations of the Patients’ Results Analyzed by Application and Human

For determining whether the application would be beneficial for the healthcare workers, a survey was carried out. The questionnaire consisted of 10 cases and contained all necessary formulas and reference ranges needed to conduct the analysis. In all cases, the diagnosis made by using the physiological and chemical approaches was precisely the same. Diagnosis of the disorder had to be made using the included reference ranges and compensation equations and based on the data presented in Table 2. The reference ranges and formulas were consistent with those used by the application. The disorder’s diagnosis was limited to its selection from the comprehensive list of possible ones found under every question. The questionnaire was completed by 62 volunteers: physicians (32.3%), medical laboratory scientists (25.8%), students of laboratory diagnostics (24.2%), medical students (14.5%), paramedics (3.2%). On average, 50.16% respondent answers were correct.

**Figure 3:**
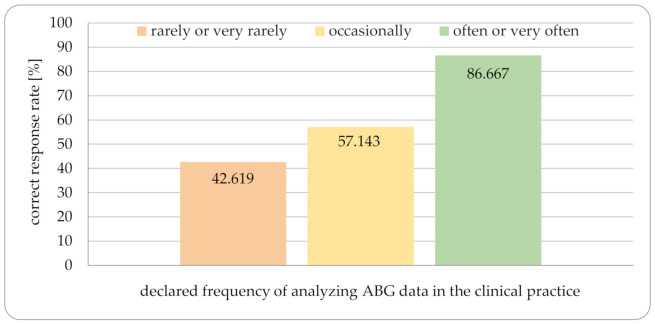
The matching of correct response rate with declared frequency of analyzing ABG data in the clinical practice.

**Figure 4:**
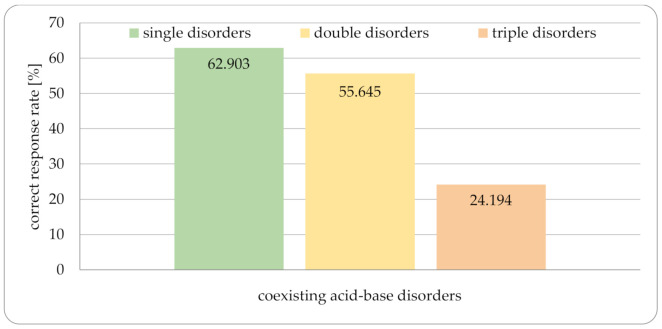
Comparison of correct response rate and number of coexisting Acid-Base disorders presented in the questionnaire.

Not surprisingly, the percentage of correct answers increased with the declared frequency of analyzing ABG data in practice. Respondents performing analyses rarely or very rarely gave fewer correct answers than those who performed them often or very often (Figure 3). The same tendencies from the 1980s [16] were also shown in our survey although the ability to analyze triple disturbances was slightly higher than in the mentioned publication (Figure 4). The questionnaire also contained one case where the ABG data were incorrect—only 41.9% of respondents noticed this. All the cases shown in Table 2 were tested by application and gave accurate results; in case no. 5, the albumin level was very low, so the best results were displayed using approaches with corrected anion gap.

## 4. Discussion

### 4.1. Overview

According to our survey results, people who do not analyze ABG daily are often mistaken in their diagnoses. The tool we created can be an excellent support for such people by speeding up confirmation of the analyzed data’s accuracy and double-checking that the analysis was carried out correctly.

We believe that the ABG Assistant can be a valuable tool for healthcare workers. As our survey results showed, this application can be helpful, especially for people who rarely analyze ABG. Our program, which analyzes the entered data through many approaches, forces such people to analyze the data in depth. On the other hand, for people who deal with ABG analysis daily, this program will be a perfect tool for quickly checking their analyses. Instead of laboriously calculating the accuracy of ABG data using the Henderson–Hasselbalch formula, our application will show whether the results are consistent.

Additionally, by obtaining a different diagnosis, the user gets a clear signal that such a case needs deeper consideration and analyses where the difference in a patient’s condition’s assessment came from. Although the ABG Assistant is not flawless, it correctly analyses more cases than the compared applications and demonstrates educational potential. The user can compare Acid-Base disorder diagnosis with the one provided by the application. Moreover, it is not important whether the chemical or physiological method is used daily—this program is useful in both cases. We hope that our publication will also stimulate discussion on the quality of analyses performed by mobile applications that was initiated by Nicholson and Corbridge [31]

The Acid-Base disorder can be an adverse prognostic indicator. Although the detection of simple disorders is not a problem, according to our survey, the detection of mixed disorders is more difficult. They do not always manifest themselves in an obvious set of symptoms. Therefore, ABG parameters can be used as biomarkers. Disease entities in which Acid-Base disorders are an integral part of diagnostic criteria can serve as a good example. One of such examples is ketoacidosis. If it occurs with a simple disorder, the diagnosis is not a problem. However, in some cases, as in the ketoalkalosis the misdiagnosis of Acid-Base disorder translates into the risk of misdiagnosis of the underlying disease. This is why our application significantly increases predictability and thus fits into the Predictive, Preventive and Personalized Medicine (PPPM) ideology, to which increasing attention is being drawn [32,33,34,35,36].

Naturally, the analysis of the Acid-Base disorders is essential in diagnosing a multitude of cases such as systemic ischemia, infections of lungs, inflammations or cancer. It also affects the patient’s treatment, for example, in birth asphyxia, where acidosis can alter the concentration of potassium in the blood [37].

### 4.2. Comparison of Available Applications

The automation of ABG interpretation is not a new idea. The first programs helping in ABG analysis were created as early as the 1970s [16]. Nonetheless, since then there has been significant technological development: computing power, once only available on personal computers, has become widely available on smartphones, which are found in the majority of societies. Research into ABG analysis also has developed: concepts, reference ranges, formulas, and even analyzed parameters are changing. During application development, we tried to include the most modern Acid-Bases analysis approach possible and use the widespread availability of smartphones to create a new mobile tool. Naturally, many applications have been developed that try to introduce newer solutions; currently, one of the most used applications for ABG analysis is MD Calc [38]. However, in our opinion, it has some limitations. Firstly, it diagnoses using only bicarbonate, while our application permits the use of SBE, calculates SBE, or analyzes data simultaneously using bicarbonate and SBE. Another limitation is the need to enter the value of albumin to obtain the analysis. Sometimes the albumin test has not been performed, or the results will be available later. In such a scenario, our application allows ABGs to be analyzed using the available data without correcting the anion gap for the influence of Alb. Tracking the albumin correction’s influence is also very simple in ABG Assistant as the analyses obtained by all methods are compiled on one card. However, the most important disadvantage of MD Calc is an incorrect analysis of some cases, which is a common problem among ABG evaluating applications, as noted by T. Nicholson [31]. The mentioned publication cites the case with the following parameters: pH, 7.4; PaCO_2_, 40 mm Hg; HCO_3_^−^, 24 mEq/L; AG, 21 (Na, 145 meq/L; Cl, 100 meq/L); Alb, 40 g/L. Regardless of the analysis method––physiological, chemical, or physicochemical––evaluation should indicate high anion gap metabolic acidosis with metabolic alkalosis. The ABG Assistant proposes just such a diagnosis for the presented data. Unfortunately, the analysis by MD Calc gives “Primary Metabolic Alkalosis, with: Appropriately Compensated by Respiratory Acidosis”. Even worse, it presents the same analysis for results that are within the reference ranges and do not represent any Acid-Base disturbance: pH, 7.4; PaCO_2_, 40 mm Hg; HCO_3_^−^, 24 mEq/L; AG, 11 (Na, 140 meq/L; Cl, 105 meq/L); Alb, 40 g/L.

### 4.3. Problematicity of Assessment

As mentioned earlier, the diagnosis of ABG creates many problems; of course, triple disorders cause the most significant diagnostic difficulties. However, even necessary activities such as checking the correctness of results were performed by a small proportion of respondents. Similar conclusions can be drawn from Schreck et al. [16], so the trend continues despite the passage of more than 30 years. As one can see from our survey, people analyzing ABG often or very often have interpreted most of the results correctly because of their experience. However, Acid-Base analysis is used by healthcare workers of various professions and specializations, including ones who use it less often, and for them, our application may have the highest value.

Unlike people, the application does not compare ABGs with a medical history and physical examination. Still, the program may point out possible solutions that a medical staff could have overlooked, even the presence of a complex disorder. Such analysis is carried out using a physiological and chemical approach, both with and without using a corrected anion gap. The use of many approaches to data analysis is beneficial because there is no consensus about which method is the best for analyzing ABGs. Some scientists try to emphasize the superiority of different approaches, while others ensure their comparability [2,3,39]. Many cases in which the analysis depends on used concepts and formulas can be mentioned. The following case may serve as an example:

pH: 7.05, PaCO_2_: 15 mmHg, sHCO_3_^−^: 4 mmol/L, SBE: −24 mmol/L, Alb 20 g/L, AG 29 mEq/L. 

The developed algorithm returned the following results:

(a) physiological approach: high anion gap metabolic acidosis
(b) physiological approach with AGc: high anion gap metabolic acidosis, possible additional metabolic alkalosis.
(c) chemical approach: high anion gap metabolic acidosis, probably with respiratory alkalosis.
(d) chemical approach with AGc: high anion gap metabolic acidosis, probably with respiratory alkalosis, possible additional metabolic alkalosis.

As one can see, there is no match between the results of the analyses. Such an example emphasizes that many cases of Acid-Base disorders are challenging to analyze. Therefore, using only one approach for an ABG analysis can sometimes lead to a wrong diagnosis. In cases like this, our program displays information about the low compliance of the analyses, thus giving the physician a clear signal that such patients should be given additional attention when being diagnosed.

During the analysis of the results, an additional aspect is worth noticing: our application displayed the results using the word “probably”. Indeed, an ABG analysis is complicated not only because of the multitude of formulas but also because of difficulties in confirming their correctness [4]. Therefore, the certainty of diagnosis decreases with the number of coexisting disturbances; the ΔAG/ΔHCO_3_^−^ ratio is also a parameter with many false-positive or negative results. Even in the ABG analysis literature, it is possible to spot vague statements like “actual HCO_3_^−^ is almost like predicted” [40]. As one can notice, the formulation “almost” in the context of ABG analysis is too often overused as an indication of a confirmation of the hypothesis. Such results can and should be treated as uncertain when they are on the border of two solutions. To make the diagnosis of disorders more realistic in our program, we changed the display of non-obvious, borderline results. Such cases are displayed as “probable” if the result exceeds the calculated compensation range but does not exceed it by 5%. The program gives the additional information that the result is less dependable and must be confirmed. The physician has to decide which option is more consistent with the patient’s condition. 

### 4.4. Limitations

Of course, the program we wrote has several restrictions. As mentioned earlier, the main one is the analysis of the ABG results without additional information about the patient’s clinical condition. Although the most common causes of the underlying disorders are listed in the “information” panel. The second is the small number of analyzed parameters. Therefore, the application cannot recognize some diseases. It can easily cope with the analysis of Acid-Base disorders occurring during diabetic ketoacidosis (DKA) or DKA with normal pH. Still, when the program analyses DKA with alkalemia—with a pH above 7.44 and AG lower than 25 mEq/L, the program can display only metabolic alkalosis. A physician carefully analyzing such results can notice the possibility of metabolic alkalosis and coexisting high anion gap metabolic acidosis present in DKA with alkalemia [41]. Currently, no program meets all the requirements for an ABG analysis. In the future, however, we would like to create a new, more extensive tool to analyze more laboratory tests results concerning ketones, lactate, glucose, osmolality, creatinine, and urine, and possibility set personal reference ranges for each parameter. The use of individual reference ranges will allow for a more personalized analysis of the entered data and thus act in accordance with PPPM.

## 5. Conclusions

In medical practice, mobile applications and computer programs might not achieve as detailed analysis as that made by specialists for many more decades. An application cannot be a substitute for an accurate, complete diagnosis. Nevertheless, based on our survey results, healthcare workers who analyze blood gases less often in daily clinical work have considerable problems, especially when it comes to mixed disorders. Thus, our study group developed an application called “ABG Assistant” to aid healthcare workers in blood gas interpretation. We hope that it will have an impact on the progress of predictive diagnostics and personalized treatment. Using this application should help draw attention to aspects that some overworked specialists may not notice or forget about: diagnosing double and triple disorders, correcting the anion gap, and verifying data. 

## Figures and Tables

**Table 1:** Comparison of results from literature and application based on the same data.

Cases			Analyzed Parameters					
			pH	PaCO_2_ (mm Hg)	HCO_3_^−^ (mmol/L)	SBE (mmol/L)	AG (mEq/L)	Alb (g/L)
No 1	values:		7.39	39	22	-	19	-
	publication diagnosis:		metabolic acidosis and metabolic alkalosis					
	application diagnosis:	physiological approach	high anion gap metabolic acidosis and metabolic alkalosis					
		chemical approach	high anion gap metabolic acidosis and metabolic alkalosis					
No 2	values:		7.41	41	24	-	19	-
	publication diagnosis:		metabolic acidosis and respiratory alkalosis					
	application diagnosis:	physiological approach	high anion gap metabolic acidosis and metabolic alkalosis					
		chemical approach	high anion gap metabolic acidosis and metabolic alkalosis					
No 3	values:		7.36	22	11	-	25	-
	publication diagnosis:		metabolic acidosis and respiratory acidosis					
	application diagnosis:	physiological approach	respiratory alkalosis and high anion gap metabolic acidosis, possible additional metabolic alkalosis					
		chemical approach	respiratory alkalosis and high anion gap metabolic acidosis, possible additional metabolic alkalosis					
No 4	values:		7.27	44	19	-	16	-
	publication diagnosis:		metabolic acidosis and respiratory acidosis					
	application diagnosis:	physiological approach	high anion gap metabolic acidosis and respiratory acidosis, possible additional metabolic alkalosis					
		chemical approach	high anion gap metabolic acidosis and respiratory acidosis, possible additional metabolic alkalosis					
No 5	values:		7.6	45	51	-	14	-
	publication diagnosis:		metabolic alkalosis and respiratory alkalosis					
	application diagnosis:	physiological approach	metabolic alkalosis and respiratory alkalosis					
		chemical approach	metabolic alkalosis and respiratory alkalosis					
No 6	values:		7.42	59	36	-	7	-
	publication diagnosis:		metabolic alkalosis and respiratory acidosis					
	application diagnosis:	physiological approach	respiratory acidosis and metabolic alkalosis					
		chemical approach	respiratory acidosis and metabolic alkalosis					
No 7	values:		7.47	23	16	-	44	-
	publication diagnosis:		chronic respiratory alkalosis, high anion gap metabolic acidosis, and metabolic alkalosis.					
	application diagnosis:	physiological approach	chronic respiratory alkalosis high anion gap metabolic acidosis and metabolic alkalosis					
		chemical approach	chronic respiratory alkalosis high anion gap metabolic acidosis and metabolic alkalosis					
No 8	values:		7.01	26	6	-	43	-
	publication diagnosis:		high anion gap metabolic acidosis, respiratory acidosis, and metabolic alkalosis					
	application diagnosis:	physiological approach	high anion gap metabolic acidosis and respiratory acidosis, possible additional metabolic alkalosis					
		chemical approach	high anion gap metabolic acidosis and respiratory acidosis, possible additional metabolic alkalosis					
No 9	values:		7.06	28	8	-	10	23
	publication diagnosis:		high anion gap metabolic acidosis and respiratory acidosis					
	application diagnosis:	physiological approach	normal anion gap metabolic acidosis and respiratory acidosis					
		physiological approach with AGc	high anion gap metabolic acidosis and respiratory acidosis, possible additional normal anion gap metabolic acidosis					
		chemical approach	normal anion gap metabolic acidosis and respiratory acidosis					
		chemical approach with AGc	high anion gap metabolic acidosis and respiratory acidosis, possible additional normal anion gap metabolic acidosis					

**Table 2:** Characteristics of the cases which were analyzed in the survey.

Cases		Parameters					
		pH	PaCO_2_ (mm Hg)	HCO_3_^−^ (mmol/L)	SBE (mmol/L)	AG (mEq/L)	Alb (g/L)
No 1	values:	7.419	20	12.5	−10.79	18.5	40
	disorder:	Respiratory alkalosis and high anion gap metabolic acidosis.					
No 2	values:	7.4	40	23.95	−0.4	19	40
	disorder:	High anion gap metabolic acidosis and metabolic alkalosis.					
No 3	values:	7.423	19	12	−11.2	34	40
	disorder:	Respiratory alkalosis, high anion gap metabolic acidosis and metabolic alkalosis.					
No 4	values:	7.068	61	17	−11.44	29	41
	disorder:	Respiratory acidosis, high anion gap metabolic acidosis and metabolic alkalosis.					
No 5	values:	7.331	31.3	16	−8.75	10.5	28
	disorder:	High anion gap metabolic acidosis.					
No 6	values:	7.438	38	18	−5.42	17	46
	disorder:	Incorrect data.					
No 7	values:	7.638	28	29	7.55	10	37
	disorder:	Respiratory alkalosis and metabolic alkalosis.					
No 8	values:	7.522	27	21.4	−1.11	11	43
	disorder:	Acute respiratory alkalosis.					
No 9	values:	7.042	82	21	−8.09	8	39
	disorder:	Respiratory acidosis and normal anion gap metabolic acidosis.					
No 10	values:	7.308	52	25.2	−0.52	9.6	38
	disorder:	Acute respiratory acidosis.					

## Data Availability

The data presented in this study is contained within this article.
